# Is Race a Risk Factor for the Development of Renal Artery Stenosis?

**DOI:** 10.4061/2009/817987

**Published:** 2009-11-17

**Authors:** Ayad Jazrawi, Saba Darda, Peter Burke, Marcos Daccarett, Josef Stehlik, Shukri David, Marcel Zughaib

**Affiliations:** ^1^Division of Cardiology, Providence Hospital and Medical Center, Southfield, MI 48075, USA; ^2^Division of Cardiology, University of Utah, Salt Lake City, UT 84112, USA

## Abstract

Atherosclerotic renal artery disease is a common cause of hypertension and chronic kidney disease that may progress into end stage renal failure if not diagnosed and treated early. Renal artery stenosis (RAS) has been shown to be an independent risk factor for mortality in patients with coronary artery disease. We sought to determine whether race is an independent risk factor for developing RAS. A retrospective study was conducted including 324 patients with resistant hypertension who underwent renal angiography with or without coronary angiography. In univariate analysis, Caucasian race was associated with significant risk of RAS (OR = 2.3, *P* = .01). However, this association was no longer significant after correcting for additional clinical variables in a multivariate model (OR = 1.5, *P* = .07). There was a strong association between smoking and RAS (OR 2.0, *P* = .02). We conclude that traditional risk factors, especially smoking, rather than race, are the most important predictors of RAS development.

## 1. Background

Atherosclerotic renal artery disease is a common cause of hypertension and chronic kidney disease. End-stage renal disease may result from hemodynamically significant renal artery stenosis (RAS) unless recognized and treated early [1–3]. RAS has been shown to be an independent risk factor for mortality in patients with coronary artery disease (CAD). Patients with atherosclerotic RAS and CAD have twice the risk of mortality even when coronary revisualization is performed [4]. In addition to age, history of atherosclerosis elsewhere, and smoking, Caucasian race has been proposed as a risk factor for development of RAS [5, 6]. We sought to investigate whether race is indeed an independent risk factor for RAS.

## 2. Methods

This was a retrospective analysis. The study group included patients who were referred for nonemergent coronary or peripheral angiography between June 2007 and May 2008, who also had hypertension resistant to medical therapy. Resistant hypertension was defined as a systolic blood pressure greater than 140 mmHg while on at least two antihypertensive medications. Patients with fibromuscular dysplasia, glomerular filtration rate (GFR) <30 mL/min/1.73 m^2^, and iodine allergy were excluded from the study. Patients with glomerular filtration rate (GFR) <30 mL/min/1.73 m^2^ were excluded since the purpose of the screening studies was to diagnose RAS prior to developing end stage renal disease. Patients with iodine allergy were excluded since they were pretreated with steroid therapy. De Matteo and May have suggested that glucocorticoids can cause renal artery vasodilatation and therefore may underestimate the degree of stenosis [7]. 

Patients who met the inclusion criteria for RAS screening per ACC/AHA guideline [8] underwent renal artery angiography to rule out RAS. 

RAS was defined as >70% luminal stenosis of the main renal artery of either or both the kidneys. 

Continuous variables were compared using *t*-test. Chi-square test was used for comparison of categorical variables. Association between race and RAS was explored with both univariate analysis and multivariate logistic regression analysis. Multiple clinical variables that included age, gender, race, history of diabetes mellitus, smoking, dyslipidemia, peripheral vascular disease (PVD), and CAD were evaluated as independent predictors of RAS.

Approval for the study was obtained from the hospital's institutional review board (IRB).

## 3. Results

There were 5012 patients who underwent diagnostic coronary or peripheral angiographic procedures during the study period. Of these, 324 patients also underwent renal angiography at the same time due to persistent hypertension while on at least two antihypertensive agents. In one instance, the RAS was determined to be due to FMD and this patient was excluded from the analysis. Therefore, the study group included 323 patients. The mean age was 65 ± 10 years, 153 (47%) patients were male, 193 (59%) were Caucasian, and 130 (41%) were non-Caucasian. Atherosclerotic RAS was diagnosed in 64/323 (20%) patients. The baseline clinical characteristics were comparable between the Caucasian and non-Caucasian patients except for higher age of Caucasian patients and higher prevalence of smoking in non-Caucasian patients (Table 1).

**Figure 1:**
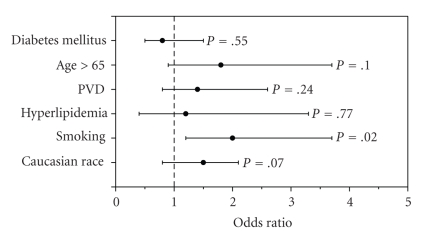
Results of multivariable analysis of the risk of renal artery stenosis.

In univariate analysis (Table 2), age >65 years, history of PVD and Caucasian race was associated with higher prevalence of RAS. To correct for possible confounding caused by differences of baseline characteristics in the two groups, we carried out a multivariate logistic regression analysis. In the multivariate model, the association between race and RAS was no longer significant (OR 1.5, 95% CI; 0.8–2.1 *P* = .07). Smoking remained as the only characteristic independently associated with RAS (Table 3, Figure 1).

## 4. Discussion

Renovascular disease is the most common cause of potentially reversible secondary hypertension [9]. In the U.S. general population, the prevalence of RAS is estimated to be 0.13% but this incidence is much higher in patients with hypertension who also have other risk factors for cardiovascular disease [10]. Several studies have reported that RAS appears to be more prevalent in Caucasian than in non-Caucasian patients [5, 9]. As a result, it has been suggested that non-Caucasian hypertensive patients should not undergo screening for RAS [11]. Other reports have questioned the association between race and RAS [12]. The limitation of many previous studies has been the use of duplex sonography for RAS screening. Although it is one of the noninvasive modalities that is primarily used to screen for RAS, it is highly operator dependent and technically demanding [13]. Catheter angiography has a higher accuracy in determining the severity of RAS [14]. 

In our study we defined RAS as >70% stenosis, since this degree of obstruction is believed to be associated with higher mortality [15].

We took advantage of our diverse patient population and an established program of screening renal angiography in patients perceived to have high probability of atherosclerotic RAS, as recommended by ACC/AHA guidelines for the Management of patients with peripheral arterial disease [8]. We show that, although in univariate analysis Caucasian race appeared to be a risk factor for RAS development, this was no longer true in our multivariable model that corrected for differences in baseline characteristics. In our model, smoking remained the only statistically significant predictor in RAS, a finding consistent with other reports [3, 16]. 

The study limitations include its retrospective nature and an assumption that there was not a selection bias in performing screening renal angiograms in the study group. The study sample size was relatively small. 

## 5. Conclusion

Our study demonstrates that traditional risk factors, especially smoking, remain closely associated with atherosclerotic renal artery stenosis. The presence of RAS should be considered in patients with resistant hypertension regardless of race.

## Figures and Tables

**Table 1:** Clinical characteristics in patients of Caucasian and non-Caucasian race.

	Caucasian race (*N* = 193)	Non-Caucasian race (*N* = 130)	*P*-value
Age (years)	70	66	.02
Gender, female	27.86 (90)	24.77 (80)	.07
Hypertension	100 (193)	100 (130)	.97
Diabetes mellitus	25.39 (82)	20.42 (66)	.20
Hyperlipidemia	54.18 (175)	33.13 (107)	.7
Peripheral vascular disease	31.37 (101)	18.01 (58)	.19
Congestive heart failure	9.38 (30)	6.88 (22)	.71
Cerebrovascular accident	12.69 (41)	9.60 (31)	.58
Smoking	21.36 (69)	10.22 (33)	.05
Serum creatinine (mg/dl ± SD)	1.6 ± 0.3	1.7 ± 0.2	.28
Renal artery stenosis	75 (48)	25 (16)	.005

**Table 2:** Univariate analysis of the risk of renal artery stenosis.

Parameter	Odds ratio	95% confidence interval	*P*-value
Diabetes mellitus	0.8	0.5–1.4	.48
Age >65 years	2.2	1.1–4.3	.02
PVD	1.7	1.0–3.0	.05
Hyperlipidemia	1.9	0.7–5.1	.19
Caucasian Race	2.3	1.3–4.4	0.01

**Table 3:** Multivariate analysis of the risk of renal artery stenosis.

Parameter	Odds ratio	95% confidence interval	*P*-value
Diabetes mellitus	0.8	0.5–1.5	.55
Age (>65 years versus <65 years)	1.8	0.9–3.7	.10
PVD	1.4	0.8–2.6	.24
Hyperlipidemia	1.2	0.4–3.3	.77
Smoking	2.0	1.2–3.7	.02
Caucasian race	1.5	0.8–2.1	.07
